# Tuning Liposome Membrane Permeability by Competitive Peptide Dimerization and Partitioning-Folding Interactions Regulated by Proteolytic Activity

**DOI:** 10.1038/srep21123

**Published:** 2016-02-19

**Authors:** Seng Koon Lim, Camilla Sandén, Robert Selegård, Bo Liedberg, Daniel Aili

**Affiliations:** 1 Centre for Biomimetic Sensor Science, School of Materials Science and Engineering, Nanyang Technological University, Research Techno Plaza, 6th storey XFrontiers block, 50 Nanyang Drive, 637553 Singapore; 2 Division of Molecular Physics, Department of Physics, Chemistry and Biology, Linköping University, 581 83 Linköping, Sweden

## Abstract

Membrane active peptides are of large interest for development of drug delivery vehicles and therapeutics for treatment of multiple drug resistant infections. Lack of specificity can be detrimental and finding routes to tune specificity and activity of membrane active peptides is vital for improving their therapeutic efficacy and minimize harmful side effects. We describe a *de novo* designed membrane active peptide that partition into lipid membranes only when specifically and covalently anchored to the membrane, resulting in pore-formation. Dimerization with a complementary peptide efficiently inhibits formation of pores. The effect can be regulated by proteolytic digestion of the inhibitory peptide by the matrix metalloproteinase MMP-7, an enzyme upregulated in many malignant tumors. This system thus provides a precise and specific route for tuning the permeability of lipid membranes and a novel strategy for development of recognition based membrane active peptides and indirect enzymatically controlled release of liposomal cargo.

A large number of natural and designed peptides have the ability to remodel and perturb the structure and permeability of cell membranes by triggering membrane disruption^1^,^2^, fusion^3^,^4^, or translocation^5^,^6^. These so-called membrane active peptides have a large structural, functional and compositional diversity, but tend to be amphiphatic and rich in positively charged amino acids. They also readily accumulate at the lipid membrane interface^7^,^8^, which can lead to membrane partitioning or cellular internalization. Membrane active peptides are often unstructured in solution but adopt a defined secondary structure when associating to a lipid membrane^9^. One important class of membrane active peptides is the antimicrobial peptides (AMPs). They are an integral part of the first line of host defence against infections in a wide variety of organisms and show broad-spectrum antibiotic activity^10^. AMPs have been studied for centuries but the occurrence of multi-drug resistance of pathogens to conventional antibiotics, including carbapenem-resistant Enterobacteriaceae^11^,^12^, have lead to an overwhelming need for new antibiotics and consequently a renewed interest in AMPs^2^,^13^,^14^. AMPs can demonstrate a significant effect on both Gram-positive and Gram-negative bacteria, as well as on fungi, enveloped viruses, parasites as well as cancerous cells and are hence potentially highly attractive candidates for treatment of antibiotic-resistant bacterial infections^15^.

Membrane active peptides can interact with lipid membranes in a multitude of ways, leading e.g. to perturbation of the membrane either by formation of transmembrane pores (barrel-stave or toroidal pores), or by a surface-associated carpet mechanism^1^,^2^,^8^. They, however, typically lack specific high affinity interactions with particular membrane components^9^, and AMPs hence show minimal inhibitory concentrations in the micromolar range^1^. The initial peptide-membrane association is typically the rate-limiting process but can be accelerated by peptide lipidation^16^. Lipidation increases their affinity for lipid membranes, which potentially can improve their therapeutic efficacy. The modifications of short peptides with lipids have turned otherwise non-membrane active peptides into AMPs demonstrating both antifungal and antiviral properties^17^,^18^. Significant improvement in activity by lipidation of the glycopeptide antibiotic vancomycin on resistant bacterial strains has also been reported^19^. Lipidation of AMPs can, however, dramatically decrease their water solubility and increase toxicity^15^. A more suitable strategy to increase AMP membrane affinity and selectivity would hence be to trigger specific and high affinity binding of the peptides to species already present in the lipid membrane. This is further motivated by the observations that AMPs, in addition to their bactericidal properties, can also demonstrate adverse toxic effects on healthy non-pathogenic cells and exhibit undesirable and significant haemolytic activity^15^. Finding routes to tune and increase the specificity and activity of both natural AMPs and designed membrane active peptides is consequently critical to avoid potential toxicity and improve their therapeutic efficacy. Moreover, designed membrane active peptides that offer target specificity and tunable activity could also be of great interest for liposome-mediated drug delivery, drug release, and gene transfection^20^. Since membrane active peptides can modulate the integrity of lipid membranes they could potentially also be exploited for regulating the release of liposome-encapsulated drugs. Possibilities to specifically tune and trigger the release of liposome-encapsulated drugs would enable better spatiotemporal control over the release and thus lowering of the required therapeutic dose.

Here, we describe a *de novo* designed membrane active helix-loop-helix peptide (JR2KC) that partition into zwitterionic lipid membranes when specifically and covalently anchored to head-group functionalized lipids present in the membrane. Anchoring and subsequent membrane partitioning triggers a structural transition of the peptide from random coil to α-helical conformation and pore formation as schematically outlined in Fig. 1. The extent of pore formation can be dynamically tuned by varying the number of anchoring moieties or by introducing a complementary helix-loop-helix peptide (JR2E) that is designed to heterodimerize with JR2KC and fold into four-helix bundles^21^. Moreover, we show that the inhibiting activity of JR2E can be controlled enzymatically by the matrix metalloproteinase MMP-7. Upregulation of MMP-7 is associated with the progression of a range of malignant tumours and is thought to be involved in cancer metastasis and various inflammatory processes^22^. JR2E contains two specific recognition sites for MMP-7, at positions 11-Ala-|-Leu-12 and 26-Ala-| Gln-27, and proteolytic cleavage of JR2E results in a truncated peptide^23^, that is unable to heterodimerize with JR2KC. MMP-7 thus reactivates the membrane permeabilizing effect of JR2KC. Here, we thus show a novel recognition based strategy for specific tuning of the permeability of lipid membranes using a *de novo* designed membrane active peptide. In addition to demonstrating a possible path for obtaining more specific designer AMPs, this system enables enzyme mediated liposomal release. The latter indicates a possible strategy for design of peptides for increasing efficacy of liposome-encapsulated anti-cancer drugs by increasing the release rate in the vicinity of tumors.

## Results

**Figure 2:**
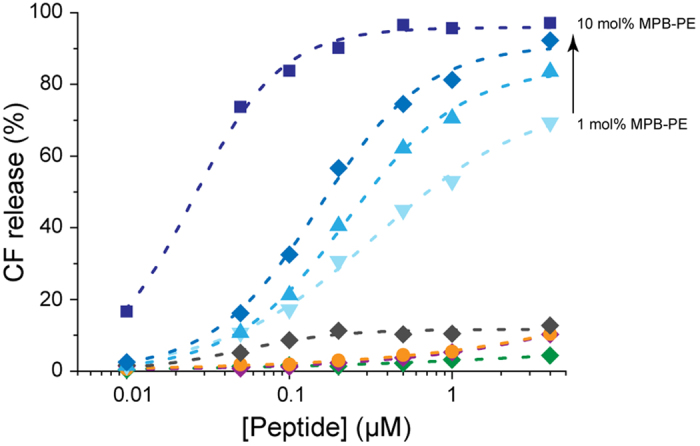
Release of carboxyfluorescein (CF) from liposomes after 30 min incubation with JR2KC (blue) using 1, 3, 5, and 10 mol% MPB-PE as indicated by the arrow. Increasing concentration of MPB-PE lead to more extensive release by JR2KC. Liposomes with 0 mol% MPB-PE (orange) show limited release, and similar to JR2K (green), oxidized JR2KC (grey), and scrambled polypeptide (purple) using liposomes with 5 mol% MPB-PE. Note, orange and purple curves overlap. All data points are average of n = 3, SD ≤ 2%.

JR2KC and JR2E were originally designed to adopt a helix-loop-helix motif and heterodimerize into four-helix bundles in solution at neutral pH values. JR2KC has an amphipathic residue distribution with a hydrophobic face and a highly charged polar face rich in lysine (Supplementary Fig. 1). The properties of JR2KC are thus reminiscent of many natural AMPs that are typically both cationic and amphipathic^14^,^24^,^25^,^26^. Exposing carboxyfluorescein (CF) loaded liposomes prepared from zwitterionic POPC to JR2KC (4 μM) showed that the peptide had limited effect on the membrane integrity and caused only minor release of CF (Fig. 2). In order to promote specific peptide membrane association maleimide head-group functionalized lipids (MPB-PE) were included at varying concentrations in the membrane. The maleimide moiety selectively reacts with the thiol group in the cysteine residue in the peptide loop region via a Michael addition, and thus covalently anchors the peptides to lipids in the membrane (Supplementary Fig. 2).

A dramatic increase in CF release was observed from liposomes prepared with as little as 1 mole percent (mol%) MPB-PE compared to liposomes lacking maleimides (Fig. 2). Less than 10% CF release was observed from POPC liposomes without maleimide anchors (0 mol% MPB-PE) in the presence of 4 μM JR2KC after 30 min incubation. In the presence of MPB-PE, the CF release was significant and highly dependent on both the amount of MPB-PE and the concentration of JR2KC. Increasing the peptide concentration from 0–4 μM resulted in faster release kinetics and more extensive release (Fig. 2 and Supplementary Fig. 3). Increasing the amount of MPB-PE in the membrane enhanced the release rates and significantly reduced the peptide concentration required to trigger CF release. In the absence of peptide the CF release for all liposome compositions was negligible (Supplementary Fig. 4). No effect on membrane integrity was obtained when replacing the cysteine residue in JR2KC by a valine, preventing the peptide from covalently binding to the maleimide moiety in MPB-PE (Fig. 2). The CF release was also abolished when oxidizing the cysteine in JR2KC to form two disulphide-linked monomers. Compared to natural AMPs, the JR2KC-mediated membrane permeabilization was thus very specific and effective, causing about 90% release in 30 min with as little as ~100 nM of the peptide. These experiments thus show that a substantial and tunable increase in membrane permeability can be obtained by selectively and site-specifically anchoring JR2KC to the membranes.

**Figure 3:**
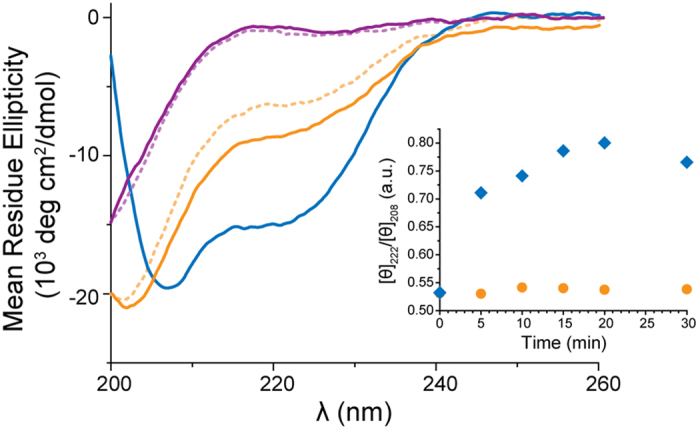
CD spectra of JR2KC (10 μM in PBS pH 7.4) in the absence (orange, dashed) and presence of POPC liposomes with 0 mol% (orange) and 5 mol% (blue) MPB-PE. The scrambled polypeptide (10 μM in PBS pH 7.4) shows no ordered secondary structure in absence (purple, dashed) nor in presence of POPC with 5 mol% MPB-PE (purple). Inset: Ratio of [θ]_222_ and [θ]_208_ for JR2KC (10 μM in PBS pH 7.4) over time in the presence of POPC liposomes with 0 mol% (orange) and 5 mol% (blue) MPB-PE.

The influence of the membrane interactions on the peptide secondary structure was investigated using circular dichroism (CD) spectroscopy. JR2KC was random coil at neutral pH, both in the absence and presence of maleimide-free (0% MPB-PE) liposomes (Fig. 3A). In the presence of liposomes with 5 mol% MPB-PE, CD spectra of JR2KC showed a significant amount of helicity, with minima at 208 and 222 nm and a mean residue ellipticity at 222 nm ([Θ]_222_) of approximately −15000° cm^2^ dmol^−1^. The initial transition from random coil to α-helical was rapid, within minutes, but the helicity continued to increase for about 10 minutes after addition of the peptide (Fig. 3B). The folding kinetics was thus on par with the rate of CF release (Supplementary Fig. 3). The folding of the peptides suggest that membrane anchoring induces partitioning of JR2KC into the hydrophobic core of the membrane, which promote induction of secondary structure via partitioning-folding coupling, as observed in many AMPs. The membrane permeabilizing effect was abolished when scrambling the primary sequence of JR2KC, keeping only the position of the cysteine residue, to form a non-amphipathic peptide (Fig. 2 and Supplementary Fig. 1). In contrast to JR2KC this peptide did not fold in the presence of MPB-PE containing liposomes (Fig. 3), clearly demonstrating that the increase in membrane permeability caused by JR2KC is both sequence-specific and folding dependent in addition to requiring anchoring of the peptide to the membrane. Dynamic light scattering (DLS) experiments showed only minor changes in liposome size (hydrodynamic radius) for liposomes with and without MPB-PE in the presence of JR2KC (Supplementary Fig. 5). The binding of the peptides to the liposomes did consequently not trigger formation of micelles, nor liposome aggregation.

**Figure 4:**
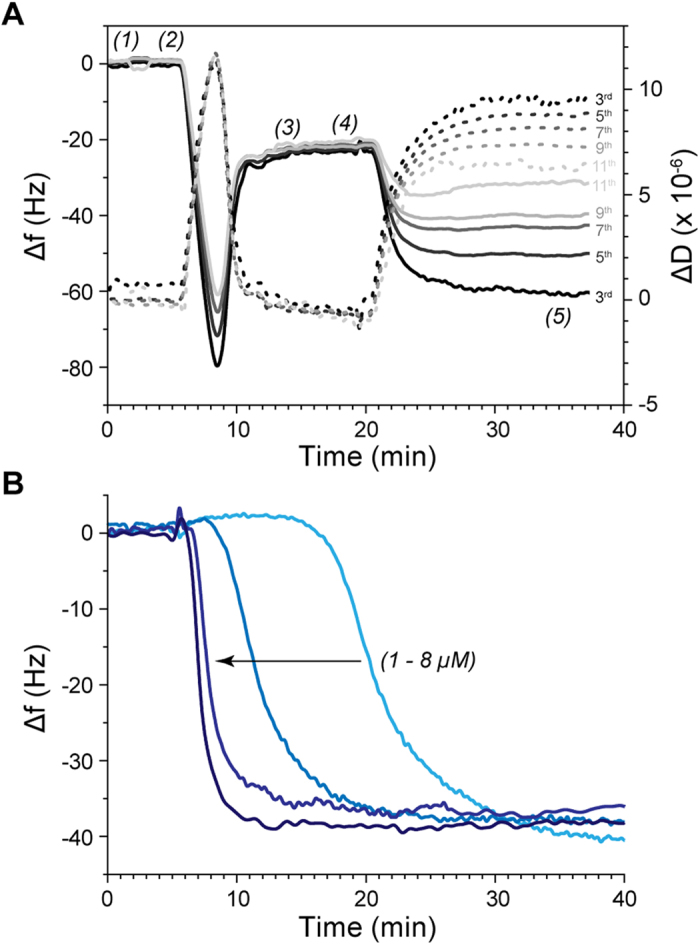
QCM-D measurement of (**A**) anchoring of 4 μM JR2KC to SLB consisting of POPC and 5 mol% MPB-PE showing Δf (solid lines) and ΔD (dashed lines) for overtones 3, 5, 7, 9 and 11 (from dark to light grey). (1) PBS buffer, (2) injection of POPC liposomes with 5 mol% MPB-PE to form a SLB, (3) rinse with PBS, (4) injection of 4 μM JR2KC and a final (5) PBS rinse. (**B**) 3rd overtone showing response in Δf after injection of 1, 2, 4 and 8 μM (from light to dark blue) JR2KC over SLB consisting of POPC and 5 mol% MPB-PE.

**Figure 5:**
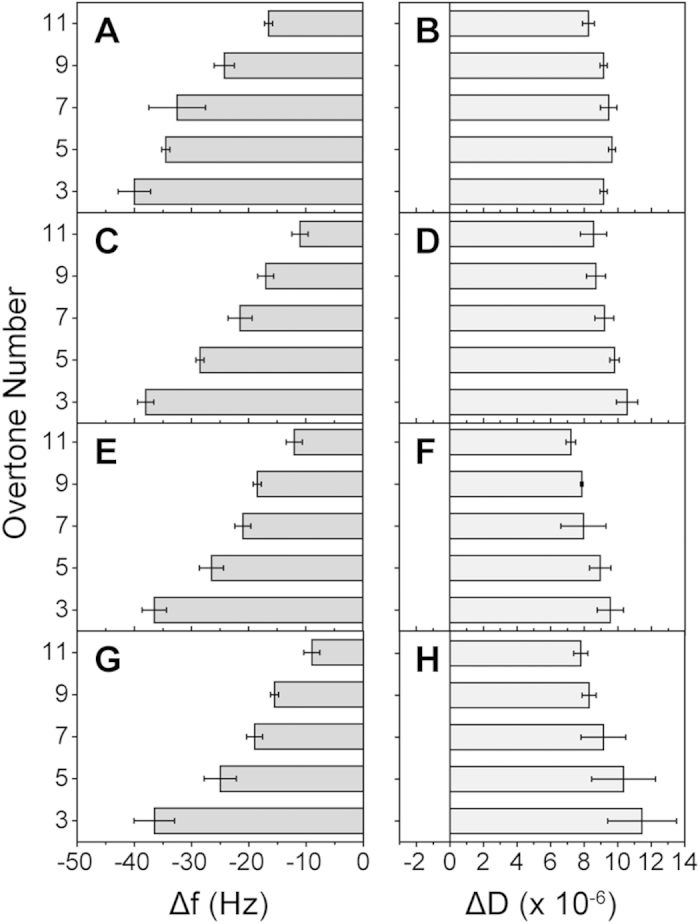
QCM-D changes in Δf and ΔD caused by JR2KC injections at concentrations of (**A,B**) 1 μM, (**C,D**) 2 μM, (**E,F**) 4 μM, and (**G,H**) 8 μM to SLB containing POPC with 5 mol% MPB-PE.

**Figure 6:**
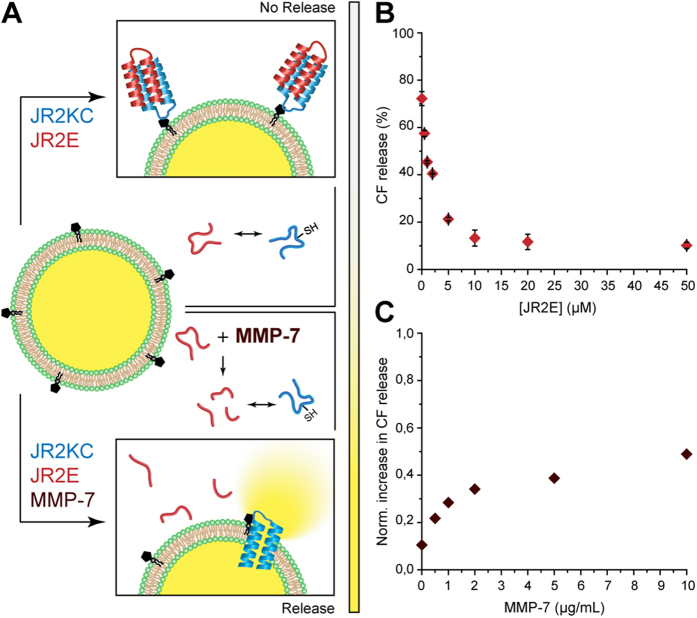
(**A**) Schematic illustration of the inhibitory effect on JR2KC upon addition of the complementary peptide JR2E (top). The two peptides fold into a helix-loop-helix motif and dimerize into a four-helix bundle. Dimerization and folding effectively prevents JR2KC from forming pores. JR2E can be proteolytically degraded by MMP-7, which restores the pore forming capacity of JR2KC (bottom). (**B**) JR2E concentration-dependent inhibition of JR2KC-triggered CF leakage from POPC liposomes with 5 mol% MPB-PE and 1 μM JR2KC, n = 3. (**C**) JR2KC triggered CF release after degradation of JR2E (50 μM) with 0, 0.5, 1, 2, 5 and 10 μg/mLMMP-7 for 2 hours, normalized with respect to the CF release of 1 μM JR2KC. Final concentrations were 1 μM JR2KC and 5 μM JR2E after addition to CF-loaded POPC liposomes with 5 mol% MPB-PE.

To further characterize the interaction of JR2KC with lipid bilayers, we examined the effect of peptide adsorption on uniform planar supported lipid bilayers (SLBs) using quartz crystal microbalance with dissipation (QCM-D). By correlating mass changes measured at different overtones, corresponding to different penetration depths, QCM-D can provide a mechanistic assessment of the peptide-mediated membrane disruption^27^,^28^,^29^. SLBs were formed by spontaneous vesicle collapse on SiO_2_ coated sensor crystals, as indicated by a frequency shift (Δf) of ~25 Hz and a dissipation shift (ΔD) close to zero. Figure 4A shows QCM-D traces of Δf and ΔD at the 3rd to 11th overtones during injection of 4 μM JR2KC over a POPC bilayer with 5 mol% MPB-PE. An initial slight increase in Δf and ΔD (position (4) in Fig. 4A) was seen, followed by a significant and rapid decrease in Δf and increase in ΔD, until a steady state was reached (Δf = −38 Hz and ΔD = 9.5 × 10^−6^ for the 3rd overtone). The large frequency shift indicates that covalent attachment of JR2KC to MPB-PE leads to further recruitment of peptides to the membrane. The initial small increases in both Δf and ΔD at all overtones indicate a certain loss of lipids caused by the association of the peptide and possibly pore formation, which makes the membrane more hydrated and less rigid. The small loss of lipids could be a consequence of increasing membrane tension upon peptide partitioning, which the planar supported lipid bilayer is unable to compensate for, in contrast to a liposome that can swell to accommodate the extra material. This event was followed by a massive decrease in Δf and increase in ΔD at all overtones, indicating that more peptides attach, which increases the total mass at the surface while decreasing the membrane rigidity. It is noteworthy that the slope of Δf and ΔD at different overtones are almost identical during the first 60 seconds of the injection of JR2KC (Fig. 4A and Supplementary Fig. 6), which indicates the initial changes in mass and viscoelastic properties are homogenous throughout the membrane, i.e. peptides likely insert into the membrane forming transmembrane pores^28^. JR2KC concentrations ranging from 1 μM to 8 μM gave similar response, Δf (~38 Hz) and ΔD (~10^−5^) (Fig. 5). The binding kinetics was, however significantly slower at lower peptide concentrations (Fig. 4B), which may indicate that a membrane concentration dependent cooperative effect is involved in the binding and insertion of the peptides. The resulting overtone-dependent Δf and ΔD shift indicate that the majority of peptides are trapped at the membrane surface, resulting in non-uniform changes of mass and viscoelasticity throughout the bilayer (Fig. 5). Importantly, membrane removal or disruption did not occur even at high peptide concentrations (8 μM), which is consistent with the DLS results, indicating that a detergent-like carpet mechanism is less likely as the mode of action. Moreover, no membrane association was seen when exposing POPC bilayers without the maleimide moiety to 8 μM JR2KC (Supplementary Fig. 7a) confirming the need for specific membrane anchoring in order for JR2KC to partition and fold. When exposing the membrane bound JR2KC to the charge-complementary peptide JR2E no further mass increase was observed (Supplementary Fig. 7b), indicating that JR2KC was not accessible for dimerization, presumably as a result of the partitioning of JR2KC into the membrane. On the other hand, when allowing JR2KC to heterodimerize with JR2E prior to addition of CF loaded MPB-PE containing liposomes, a drastic reduction in the CF release was observed (Fig. 6). At a concentration of 10 μM JR2E, the membrane-activity of JR2KC was more or less abolished. The formation of the heterodimeric four-helix bundles thus dynamically and specifically prevented the possibility for JR2KC to insert into the lipid membrane. Using different JR2E concentrations the membrane permeabilizing effect of JR2KC could be reproducibly tuned with great precision (Fig. 6B).

JR2E contains two recognition sites for MMP-7 (11-Ala-|-Leu-12 and 26-Ala-| Gln-27) and proteolytic cleavage drastically reduces the ability of JR2E to heterodimerize with JR2KC^23^. Incubation of JR2E with MMP-7 resulted in a MMP-7 concentration dependent recovery in membrane-activity of JR2KC. MMP-7 alone (1 μg/ml) did only cause a minor increase (4.6 ± 0.2%) in CF release when added to liposomes with 5 mol% MPB-PE (Supplementary Fig. 8). After exposure of JR2E to 10 μg/ml MMP-7 for 2 hours prior to mixing with JR2KC the inhibiting capacity of the peptide was reduced by about 50%. The large increase in membrane permeability of JR2KC in the presence of MMP-7 was thus a direct consequence of the hydrolytic digestion of JR2E leading to an increase in the concentration of non-dimerized JR2KC amenable to membrane partitioning.

## Discussion

The possibility to specifically bind membrane active peptides to cell-type dependent biomarkers at the cell surface could be a highly interesting strategy to improve selectivity of AMPs and other membrane active peptides of interest for therapeutic applications. In this work, we exploited the recognition of head group modified lipids to boost and tune the membrane disrupting effect of a *de novo* designed AMP-like helix-loop-helix peptide (JR2KC). Our findings indicate that recognition-based membrane anchoring could indeed be a viable and effective strategy to improve the specificity and membrane-permeabilizing activity of membrane active peptides that are limited by low efficacy and limited selectivity. The binding of the *de novo* designed membrane active peptide to thiol-reactive maleimide head group functionalized lipids (MPB-PE) specifically and significantly increased the membrane permeability by formation of pores. This 42 amino acid helix-loop-helix peptide has two approximately twenty residue-segments flanking the cysteine residue in the loop region that reacts with MPB-PE. Both segment fulfills the length requirement (>20 residues) for a peptide to form a transmembrane pore that spans the lipid bilayer^30^. Covalent anchoring of JR2KC to MPB-PE increased the effective concentration of the peptide at the membrane–water interface and reduced its conformational flexibility, facilitating partitioning and pore formation. The extent of release and kinetics could be rationally modulated by both maleimide surface concentration and peptide to lipid ratio, where increases in any of these parameters lead to faster and more extensive membrane disruption. An increase in efficacy of membrane active peptides that is dependent on the surface concentration of specific recognition moieties is a highly desirable property for future therapeutic applications.

Moreover, we show the possibilities to tune the activity of this membrane active peptide by a specific and competitive folding-dependent interaction with a complementary peptide (JR2E). The two peptides, JR2KC and JR2E, are designed to fold into a helix-loop-helix motif and heterodimerize into four-helix bundles. The heterodimerization event act as a switch that inhibits the pore forming capacity of JR2KC, presumably because hydrophobic residues are hidden in the hydrophobic core of the four-helix bundle preventing partitioning-folding coupling. The dissociation constant for the heterodimeric four-helix bundle in solution is in the micromolar range (0.02 mM) and the dimerization event displays a fast on-off rate^31^. At a concentration of 10 μM JR2E and 1 μM JR2KC, about 0.7 μM JR2KC should be available for pore formation. The concentration required for JR2E to inhibit JR2KC is thus slightly lower than what is estimated based on the dissociation constant of the free peptides. This is likely a result of a charge-charge meditated pre-concentration effect leading to an accumulation of peptides (JR2E) at the membrane interface. Since the formation of heterodimers is fully reversible, the inhibitory action of JR2E can be reverted. We have included two recognition sites for MMP-7 in the primary sequence of JR2E, located at either side of the loop-region. Proteolytic scission drastically reduces the size of the peptide making heterodimerization very unfavorable. The activity of JR2KC can thus also be modulated by the enzymatic activity of MMP-7. The indication that the pore forming capacity of designed peptides can be activated by clinically relevant biomarkers opens up for new possible strategies for tuning the specificity and efficacy of membrane active peptides in therapeutic applications and also for design of novel enzyme regulated drug delivery systems and *in vivo* sensing applications.

## Methods

### Chemicals

1-Palmitoyl-2-oleoyl-sn-glycero-3-phosphatidylcholine (POPC), 1,2-dioleoyl-sn-glycero-3-phosphoethanolamine-N-[4-(p-maleimidophenyl) butyramide] (MPB-PE) were purchased from Avanti Polar Lipids (Alabaster, USA). Other chemicals were purchased from Sigma Aldrich (Singapore) unless otherwise noted.

### Peptides synthesis

The peptides JR2KC (NAADLKKAIKALKKHLKAKGPCDAAQLKKQLKQAFKAFKRAG), JR2K (the same sequence as JR2KC but the Cys in position 22 was replaced by Val), and JR2E (NAADLEKAIEALEKHLEAKGPVDAAQLEKQLEQAFEAFERAG) were synthesized on a Symphony Quartet peptide synthesizer (Protein Technologies) using standard fluorenylmethoxycarbonyl (Fmoc) chemistry as described in more detail elsewhere^31^. The crude products were purified by reversed phase HPLC on a Ace C-8 column and identified by MALDI-TOF mass spectrometry. Scrambled JR2KC (AKLKLAAKAHKDKGIKPKALNCDAKFAKFKAKQQKRA) was purchased from GL Biochem (Shanghai, China) with a purity of >95%. The concentration of the peptides when dissolved was estimated assuming 25% water content in the lyophilized state. Oxidation of JR2KC was carried out by incubating JR2KC (2 mg/ml) in 10 mM ammonium hydrogen carbonate buffer at pH 8.5 incubated for 72 hours at room temperature. Complete oxidation was confirmed by an Ellman’s test. The oxidized peptides were diluted in MQ water, rotary evaporated and sequentially lyophilized.

### Liposome preparation

Liposomes were prepared by film rehydration followed by extrusion. Stock solutions of POPC and MPB-PE in chloroform were mixed in the following molar ratios: 100:0, 99:01, 97:03, 95:05 and 90:10. The solvent was evaporated using a gentle stream of nitrogen prior to vacuum desiccation overnight. Vesicles were formed by adding 1.0 mL of 0.1 M phosphate-buffered saline (PBS), pH 7.4 to the dried film, incubated for 20 min, followed by vortexing for 1 min. The vesicles were subsequently extruded 21 times using a Mini Extruder (Avanti Polar Lipids) through track-etched 100 nm polycarbonate membranes to form large unilamellar vesicles. For carboxyfluorescein (CF) dye encapsulation, 1.0 mL of 50 mM CF (self-quenching concentration) in 20 mM sodium phosphate, 10 mM NaCl (pH 7.4) was added to the dried lipid cakes, after which vesicle were prepared as described above. The total amount of lipid was 5 mg/mL. Unencapsulated CF was removed by gel filtration using a PD-25 column (GE Healthcare, Singapore) eluted with PBS buffer.

### Carboxyfluorescein (CF) leakage assay

Peptide-induced liposome leakage of CF was studied using a fluorescence plate reader (Infinite 200, Tecan, Austria; λ_ex_ = 485 nm, λ_em_ = 520 nm). Liposomes were diluted to 0.05 mg/mL (total lipid concentration) with PBS buffer in 96-well plate before addition of 0, 0.01, 0.05, 0.1, 0.2, 0.5, 1, 4 μM peptide. Starting with a self-quenching concentration of CF inside the liposomes, any leakage of the dye can be detected as an increase in the fluorescence intensity as a function of time. The percentage of CF release over time is calculated as 100 × (F − F_0_)/(FT − F_0_), where F_0_ is the initial fluorescence of CF before peptide addition, F is the fluorescence of CF at time interval t and FT is the total fluorescence after full release of CF upon 15 min incubation with 1% Triton X-100.

### Digestion of JR2E with MMP-7

JR2E (50 μM) was incubated with MMP-7 at concentrations of 0–10 μg/mL at 37 °C for 2 h. The reaction was quenched by heating to 80 °C for 20 min. The digested JR2E was incubated with JR2KC for 30 min at room temperature prior to addition to liposomes and analysis of CF leakage as described above.

### Circular dichroism (CD) spectroscopy

CD spectra were recorded with a Jasco J-810 spectropolarimeter (Jasco Corporation, Japan) using a 0.01 cm pathlength CD cuvette at 25 °C. CD spectra of 10 μM JR2KC dissolved in PBS buffer (pH 7.4) were recorded in the absence or presence of liposomes with 0.5 mg/ml POPC only or POPC with 5 mol% MPB-PE using a scanning speed of 50 nm/min, a resolution of 0.1 nm and a bandwidth of 1 nm. Consecutive spectra were recorded for 25 minutes after addition of peptides to liposomes. Baseline correction was performed using PBS. Three spectra of each sample were averaged.

### Quartz crystal microbalance with dissipation (QCM-D)

QCM-D experiments were performed on a Q-Sense E4 instrument (Q-Sense AB, Gothenburg, Sweden). All measurements were performed on silicon dioxide-coated sensor crystals. The crystals were cleaned with 1% w/w sodium dodecyl sulfate (SDS) solution, rinsed with water and ethanol thoroughly. After gentle drying under a stream of nitrogen, the crystals were treated using an UV ozone cleaner for 5 min. For supported lipid bilayer formation, 1 mL of 0.1 mg/mL liposome in PBS, pH 7.4, were injected at a flow rate of 100 μL/min, followed by buffer rinsing for at least 10 min. 1, 2, 4 and 8 μM JR2KC dissolved in PBS were injected at a flow rate of 100 μL/min for study of membrane immobilization and partitioning.

### Dynamic light scattering (DLS)

DLS experiments were carried out using an ALV/DLS/SLS-5022F system from ALV-GmbH, Langen Germany, using a HeNe laser at 632.8 nm with 22 mW output power. Liposome size distributions were calculated using the CONTIN 2DP routine implemented in the ALV data analysis package. All samples were filtered through a hydrophilic PVDF filter with 0.2 μm pore diameter prior to measurement. The temperature was stabilized by a thermostat bath at 21.5 °C.

## Additional Information

**How to cite this article**: Lim, S. K. *et al.* Tuning Liposome Membrane Permeability by Competitive Peptide Dimerization and Partitioning-Folding Interactions Regulated by Proteolytic Activity. *Sci. Rep.* **6**, 21123; doi: 10.1038/srep21123 (2016).

## Supplementary Material

Supplementary Information

## Figures and Tables

**Figure 1:**
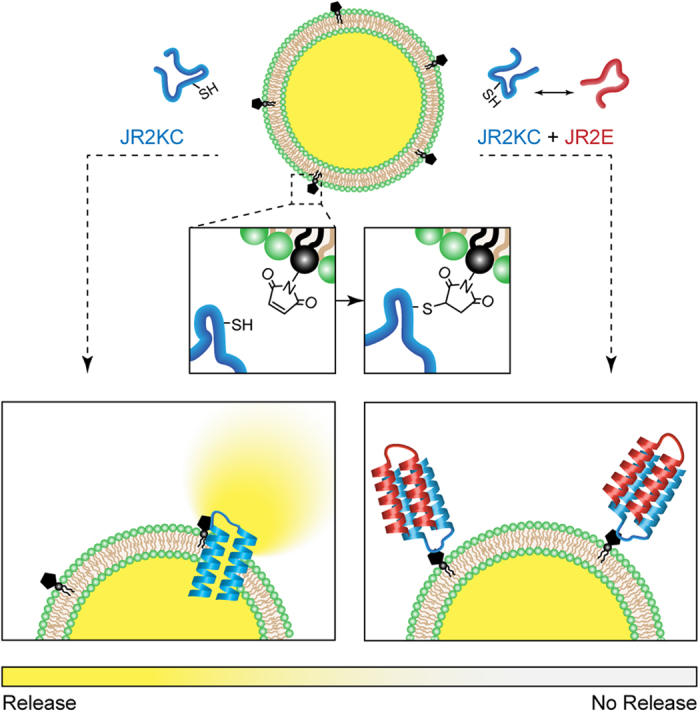
POPC liposomes with varying amounts (1–10 mol%) of a maleimide head group functionalized lipid (MPB-PE) were prepared (top). The MPB-PE lipid anchored the *de novo* designed helix-loop-helix polypeptide JR2KC to the membrane, which triggered pore formation (left). The pore formation was inhibited by addition of the charge complementary polypeptide JR2E that heterodimerize with JR2KC and folds into a four-helix bundle (right). Not drawn to scale.
