# Supplementary material for: Tuning Liposome Membrane Permeability by Competitive Peptide Dimerization and Partitioning-Folding Interactions Regulated by Proteolytic Activity
Source: Sci Rep. 2016 Feb 19;6:21123. doi: 10.1038/srep21123 (PMC4759693; doi:10.1038/srep21123)
Supplement: Supplementary Information [file srep21123-s1.docx]

Supplementary Information

**Tuning Liposome Membrane Permeability by Competitive Peptide Dimerization and Partitioning-Folding Interactions Regulated by Proteolytic Activity**

Seng Koon Lim,^1^ Camilla Sandén,^2^ Robert Selegård,^2^ Bo Liedberg,*^,1^ and Daniel Aili*^,2^

^1^Centre for Biomimetic Sensor Science, School of Materials Science and Engineering, Nanyang Technological University, Research Techno Plaza, 6th storey XFrontiers block, 50 Nanyang Drive, 637553 Singapore, ^2^Division of Molecular Physics, Department of Physics, Chemistry and Biology, Linköping University, 581 83 Linköping, Sweden

*daniel.aili@liu.se, bliedberg@ntu.edu.sg


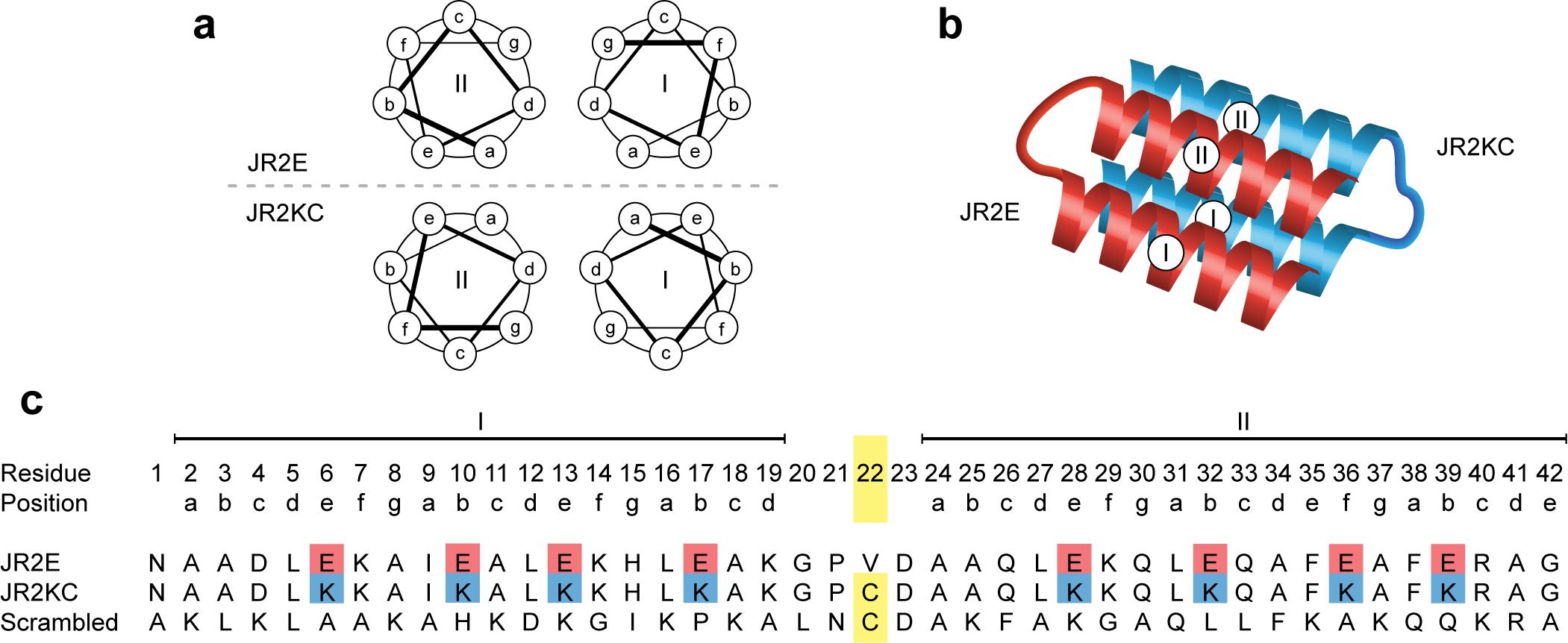


**Figure 1.** (a) Helical wheel diagram, and (b) illustration of the heterodimeric JR2E/JR2KC four-helix bundle. (c) Amino acid sequence of JR2E, JR2KC and JR2KC scrambled. The cysteine exploited for immobilization of JR2KC and JR2KC scrambled is in position 22, located in the loop region.


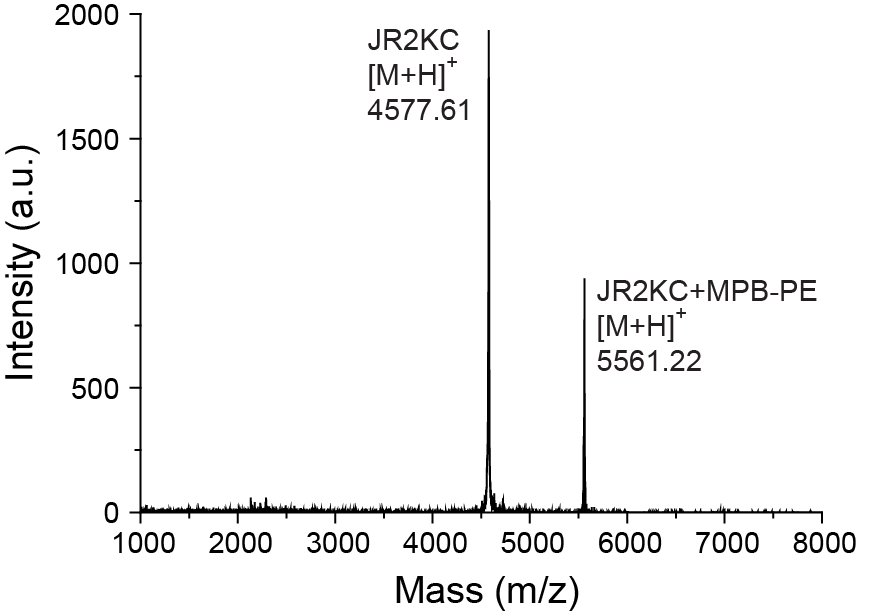


**Figure 2.** Matrix assisted laser desorption ionization time of flight mass spectrometry (MALDI-TOF) spectrum of JR2KC (0.5 mM) incubated with MPB-PE (5.0 mM) in 10 mM PB pH 7 over night. The reaction mixture was analyzed Voyager System 4212, Applied Biosystems MALDI-TOF MS with detection in the positive mode using α-cyano-4-hydroxy-cinnamic acid as matrix. Calculated mass for JR2KC [M+H]^+^: 4577.59, found: 4577.61. Calculated mass for JR2KC+MPB-PE [M+H]^+^ 5561.20, found: = 5561.29.


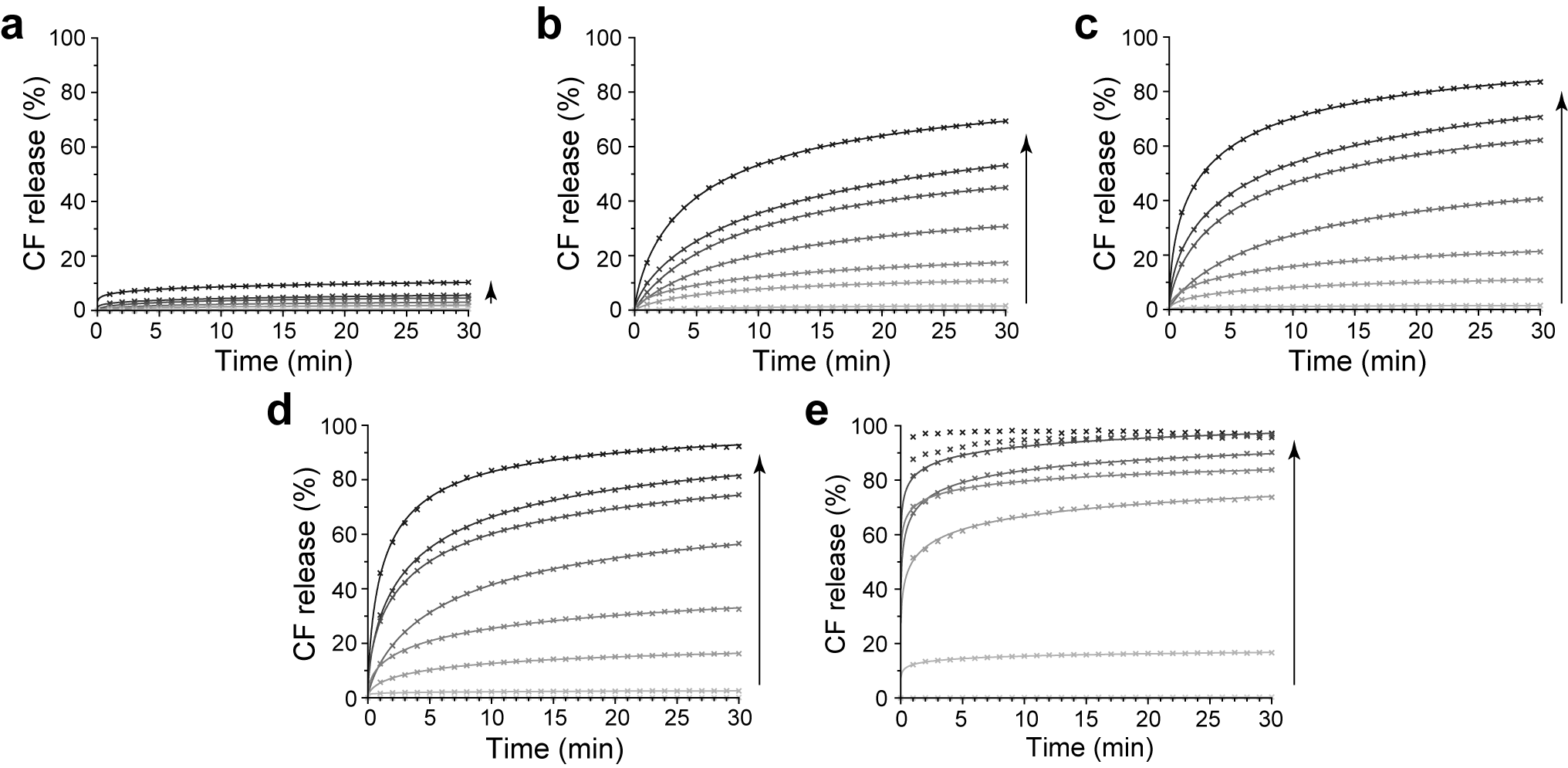


**Figure 3.** CF release at 30 min after addition of 0.01, 0.02, 0.05, 0.1, 0.2, 0.5, 1 and 4 µM JR2KC (as indicated by inset arrow from low to high peptide concentration) for POPC liposomes containing (a) 0 mol%, (b) 1 mol%, (c) 3 mol%, (d) 5 mol% and (e) 10 mol% MPB-PE. Data are fitted to an extended Langmuir model.

**
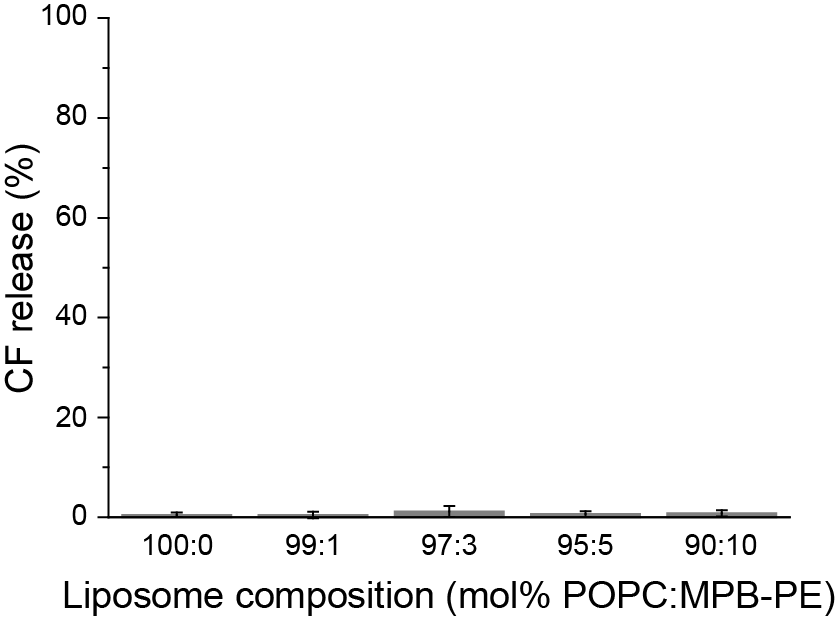
**

**Figure 4.** CF release at 30 min for liposome with different compositions of POPC-MPB-PE in the absence of JR2KC. (n=5)


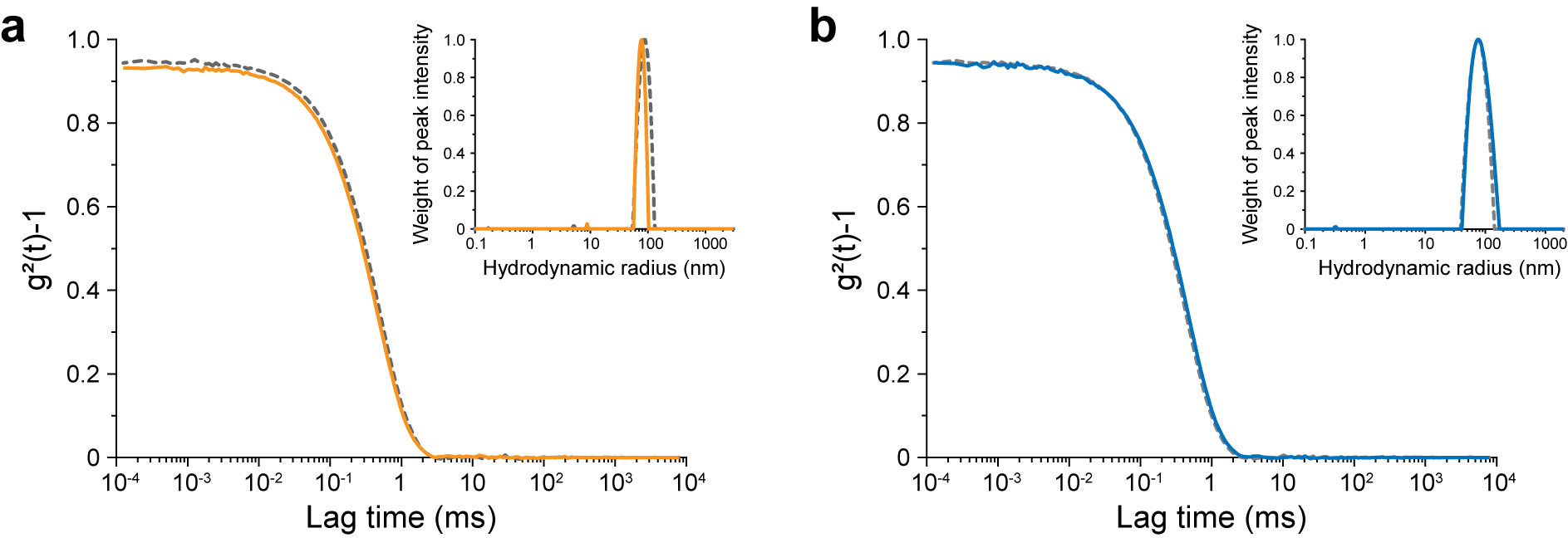


**Figure 5.** DLS correlation curves and inset size distributions obtained using the CONTIN algorithm for 10 µM JR2KC incubated with (a) POPC liposomes and (b) POPC liposomes with 5 mol% MPB-PE. Data recorded before (dashed lines) and after (solid lines) incubation with JR2KC.


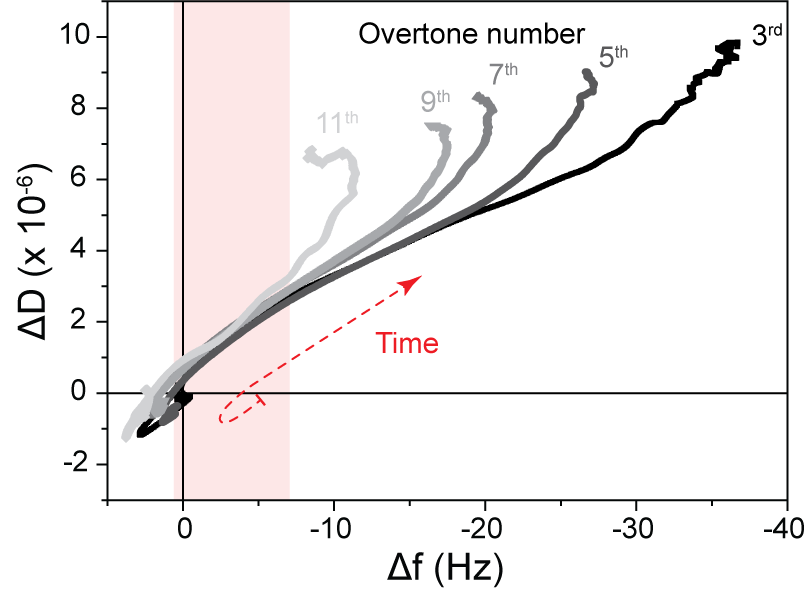


**Figure 6.** ΔD vs Δf plot from QCM-D measurement with 4 µM JR2KC added to SLB consisting of POPC with 5 mol% MPB-PE from the time point of addition of peptide to before buffer rinsing (referring to Figure 4a, point (4)-(5)). The ΔD-Δf plot indicates an initial loss of mass and simultaneously an increase in film rigidity. There are also very similar increases in both mass and viscoelasticity for all overtones during the initial peptide binding phase (pink box), suggesting that the peptides inserts in the membrane. Further additional aggregation on the membrane surface is evident from the overtone dependent increase in Δf.


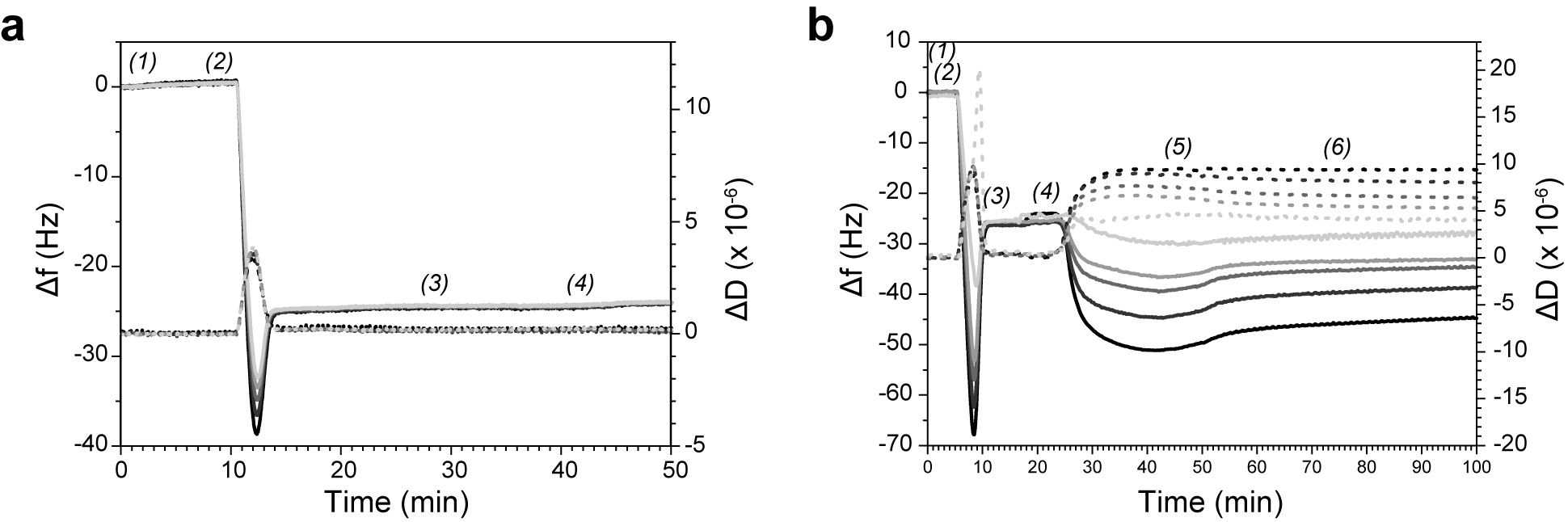


**Figure 7.** QCM-D measurement showing both ΔF (solid lines) and ΔD (dashed lines) for overtones 3, 5, 7, 9 and 11 (dark grey and sequentially to light grey). The measurement was performed by (1) setting a baseline with PBS buffer, (2) injecting liposomes consisting of liposomes to form a SLB. (a) Injection of JR2KC (8 µM) over a POPC SLB (i.e. 0 mol% MPB-PE), where (3) corresponds to the injection of JR2KC and (4) buffer rinse. Without MPB-PE, JR2KC does not anchor to the SLB. (b) Injection of JR2KC (4 µM) over a SLB with 5 mol% MPB-PE followed by injection of JR2E (8 µM) where (3) corresponds to buffer rinse, (4) the injection of JR2KC, (5) buffer rinse and (6) injection of JR2E.


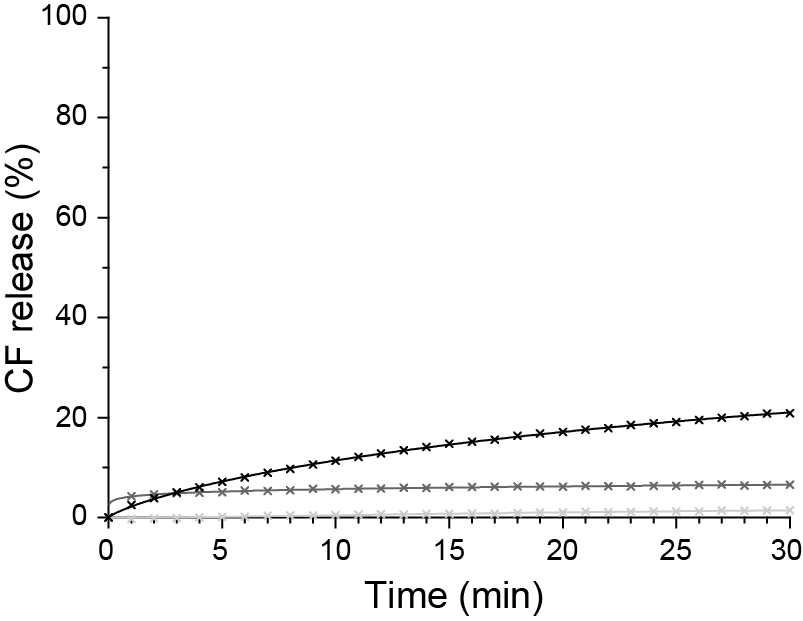


**Figure 8.** CF release after 30 min form liposomes with POPC and 5 mol% MPB-PE without any addition of MMP-7 (light grey), with 1 µg/mL MMP-7 (dark grey), and JR2KC and JR2E digested by 1 µg/mL MMP-7 (black).
